# PEGylated Mesoporous Silica Nanoparticles (MCM-41): A Promising Carrier for the Targeted Delivery of Fenbendazole into Prostrate Cancer Cells

**DOI:** 10.3390/pharmaceutics13101605

**Published:** 2021-10-02

**Authors:** Maedeh Koohi Moftakhari Esfahani, Seyed Ebrahim Alavi, Peter J. Cabot, Nazrul Islam, Emad L. Izake

**Affiliations:** 1 School of Chemistry and Physics, Science and Engineering Faculty, Queensland University of Technology (QUT), 2 George Street, Brisbane, QLD 4000, Australia; maedeh.koohi@hdr.qut.edu.au; 2 Centre for Materials Science, Queensland University of Technology (QUT), 2 George Street, Brisbane, QLD 4000, Australia; 3 School of Mechanical Engineering, Western Sydney University, Sydney, NSW 2751, Australia; s.ebrahimalavi@westernsydney.edu.au; 4 School of Clinical Sciences, Faculty of Health, Queensland University of Technology, 2 George Street, Brisbane, QLD 4000, Australia; pcabot@pharmacy.uq.edu.au; 5 School of Pharmacy, The University of Queensland, Woolloongabba, QLD 4102, Australia; nazrul.islam@qut.edu.au

**Keywords:** drug delivery systems, drug repurposing, MCM-41, mesoporous silica nanoparticle, solubility

## Abstract

Low water solubility and thus low bioavailability limit the clinical application of fenbendazole (FBZ) as a potential anticancer drug. Solubilizing agents, such as Mobil Composition of Matter Number 41 (MCM) as a drug carrier, can improve the water solubility of drugs. In this study, PEGylated MCM (PEG-MCM) nanoparticles (NPs) were synthesized and loaded with FBZ (PEG-MCM-FBZ) to improve its solubility and, as a result, its cytotoxicity effect against human prostate cancer PC-3 cells. The loading efficiency of FBZ onto PEG-MCM NPs was 17.2%. The size and zeta potential of PEG-MCM-FBZ NPs were 366.3 ± 6.9 nm and 24.7 ± 0.4 mV, respectively. They had a spherical shape and released the drug in a controlled manner at pH 1.2 and pH 6.2. PEG-MCM-FBZ were found to inhibit the migration of PC-3 cells, increase the cytotoxicity effects of FBZ against PC-3 cells by 3.8-fold, and were more potent by 1.4-fold, when compared to the non-PEGylated NPs. In addition, PEG-MCM-FBZ promoted the production of reactive oxygen species by 1.3- and 1.2-fold, respectively, when compared to FBZ and MCM-FBZ. Overall, the results demonstrate that PEG-MCM-FBZ NPs enhanced FBZ delivery to PC-3 cells; therefore, they have the potential to treat prostate cancer after a comprehensive in vivo study.

## 1. Introduction

Despite high investment in pharmaceutical research and related technologies in past decades, the failure rate of drug candidates during their phase I clinical trials is approximately 90%, and it usually takes billions of dollars and 10–15 years to develop a new drug [1]. Thus, developing suitable methods for enhancing the delivery of a drug is critical to increasing the success rate of new drug development. Recently, drug repurposing has received considerable attention from the pharmaceutical industry and research community for drug discovery [2]. Drug repurposing is a new direction in drug discovery to identify new therapeutic uses of existing drugs for treating diseases [3]. Therefore, drug repurposing is very helpful in the process of drug development when compared to the traditional de novo processes used for drug discovery [3]. In recent years, the repurposing of anthelmintics (e.g., fenbendazole (FBZ)) for the treatment of cancer has received considerable attention [4,5,6].

FBZ is used for the treatment of gastrointestinal parasites [7,8]. It is administered orally and has low permeability and solubility (0.025 µg/mL), resulting in poor bioavailability [6,9]; therefore, FBZ requires technologies to improve its water solubility and membrane permeability. Recent studies [10,11,12] have demonstrated that FBZ can cause anticancer effects. FBZ induces these effects through various mechanisms, such as (i) proteasomal inhibition; (ii) blocking of glucose uptake; (iii) disruption of microtubule function and inhibition of mitotic spindle formation, thereby leading to mitotic arrest and further resulting in apoptosis; (iv) modulation of the glycolytic pathway, and (v) P53 stabilization [10,12]. In addition, FBZ can be used with other anticancer agents, such as rapamycin, to synergize its anticancer effects [13]. FBZ is administered orally and has low permeability and solubility (0.025 µg/mL), which results in its poor bioavailability [6,9]. Therefore, the clinical use of FBZ requires improving its water solubility and membrane permeability.

Various nanocarrier-based drug delivery systems have been developed to modify the solubility of a therapeutic drug, manage its oral delivery, improve its membrane permeability/bioavailability, and minimize its side effects [6,14]. This is due to the favored features of nanocarriers, such as the small size and controlled drug release [15]. Mesoporous silica nanoparticles (MSNPs), such as Mobil Composition of Matter Number 41 (MCM), are inorganic drug carriers that consist of stable and rigid structures and demonstrate appropriate pH resistance as well as thermal and mechanical stress stability [16]. They have been demonstrated to improve the aqueous solubility and dissolution rate of drugs through the conversion of a crystalline drug into an amorphous form in their small nanopores [14,17]. The use of several characterization techniques demonstrated that MSNPs are able to improve the drugs’ properties in terms of the wettability and porosity of hydrophobic drugs [17], drug stability and hydrolysis degradation [18], solubility of sparingly soluble drugs [19], and oral delivery of hydrophobic drugs (e.g., FBZ) [20]. For these reasons, this study aimed to encapsulate FBZ as a repurposed and hydrophobic drug into MSNPs to improve its properties against prostate cancer PC-3 cells. For this purpose, a new nanoformulation of FBZ was synthesized using PEG-MCM nanocarrier to improve its solubility and, consequently, the cytotoxicity effects of the formulation against prostate cancer PC-3 cells. To characterize PEG-MCM-FBZ NPs, dynamic light scattering (DLS), scanning electron microscopy (SEM), transmission electron microscopy (TEM), Fourier-transform infrared (FTIR) spectroscopy, Brunauer–Emmett–Teller (BET), differential scanning calorimetry (DSC), and thermogravimetric analysis (TGA) measurements were used to determine their size, size distribution, zeta potential, morphology, chemical structure, specific surface area, glass transition temperature (T_g_), and thermal stability. Reverse-phase high-performance liquid chromatography (RP-HPLC) was used to quantify the amount of the drug released from PEG-MCM NPs at different time intervals. The biological effects of the formulation were evaluated using 3-[4,5-dimethylthiazol-2-yl]-2,5 diphenyl tetrazolium bromide (MTT), reactive oxygen species (ROS), cellular uptake, and cell migration assays.

## 2. Materials and Methods

### 2.1. Materials

Cetyltetramethylammonum bromide (CTAB), tetraethyl orthosilicate (TEOS), FBZ, phosphate-buffered saline (PBS), dimethyl sulfoxide (DMSO), tetraethyl orthosilicate (TEOS), D-α-tocopherol polyethylene glycol succinate (TPGS), carbonyldiimidazole (CDI), (3-Aminopropyl)triethoxysilane (APTES), sodium hydroxide (NaOH), 4′,6-diamidine-2′-phenylindole dihydrochloride (DAPI), phalloidin-FITC (fluorescein isothiocyanate), paraformaldehyde, bovine serum albumin (BSA), and triton X-100 were purchased from Merck (Castle Hill, NSW, Australia). Roswell Park Memorial Institute (RPMI-1640), trypsin–ethylenediaminetetraacetic acid (EDTA) (0.25%), fetal bovine serum (FBS), acetone, and formic acid (FA) were from Thermo Fisher Scientific (Scoresby, VIC, Australia). MTT and 2′-7′dichlorofluorescin diacetate (DCFH-DA) were purchased from Abcam (Melbourne, VIC, Australia) and PromoKine (Promocell GmbH, Germany), respectively. Cyanine5 NHS ester (Cy-5) was purchased from Tocris Bioscience, Australia. HPLC-grade acetonitrile was from RCI Labscan (Bangkok, Thailand). Deionized double-distilled water (Milli-Q water) was used in all experiments. PC-3 cells were kindly supplied by Dr. Jennifer Gunter (Queensland University of Technology, Brisbane, QLD, Australia).

### 2.2. Synthesis of MCM NPs

MCM NPs were synthesized according to the method of Talavera-Pech et al. [21] with some modifications. For this purpose, 1 g of CTAB was dissolved in 480 mL of Milli-Q water in a clean 1 L glass bottle using a stirrer (500 RPM, room temperature) to obtain a clear solution. Next, 3.5 mL of 2 M NaOH was slowly added, and the temperature was increased to 80 °C. Once at 80 °C, 6.7 mL of TEOS was slowly added to the mixture and stirred (700 RPM, 2 h). The suspension was filtered under vacuum and washed three times with Milli-Q water. The filtrate was dried overnight in an oven at 60 °C and crushed. The resulting powder was calcinated at 550 °C for 5 h in a muffle furnace (the temperature was slowly increased up to 550 °C (5 °C/min), held for 5 h, and slowly decreased (10 °C/min) to reach ambient temperature).

### 2.3. Synthesis of MCM-FBZ NPs

First, 40 mg of FBZ was dissolved in 15 mL of acetone as the loading solvent. Then, 160 mg of unwashed MCM NPs was added to the FBZ solution and stirred (300 RPM, room temperature) overnight. The organic solvent was then removed using a rotary evaporator (Laborota 4000 HB/G1, Heidolph, Germany), and FBZ-loaded MCM (MCM-FBZ) NPs were obtained.

### 2.4. Surface Functionalization of MCM with Amine Group

First, 100 mg of MCM NPs was dispersed into 10 mL of toluene and sonicated for 3 min. Next, 300 µL of APTES was added dropwise and stirred (500 RPM, 37 °C) overnight. The suspension was then centrifuged (15,000 RPM, 3 min), and the supernatant was removed. The prepared MCM-NH_2_ NPs were washed twice with ethanol and once with water using centrifugation (12,000 RPM, 3 min), and then dried in an oven at 60 °C.

### 2.5. Synthesis of PEG-MCM-FBZ NPs

TPGS-CDI was synthesized according to the method previously described by Cheng et al. [22]. For this purpose, 113.5 mg (15 mM) of TPGS and 60.8 mg (75 mM) of CDI were dissolved in 10 mL of distilled dioxane using vortex. The solution was then incubated under N_2_ environment for 24 h at room temperature while stirring (500 RPM). The solvent was removed using vacuum rotary evaporation, 5 mL of Milli-Q water was added to the resulting precipitate, and then it was freeze-dried.

To synthesize PEG-MCM NPs, 100 mg of the resulting freeze-dried TPGS-CDI was dissolved in 5 mL of Milli-Q water, to which 100 mg of MCM-NH_2_ NPs was added and mixed overnight at room temperature on a stirrer (500 RPM). The mixture was then centrifuged (12,000 RPM), and the supernatant was removed. The formed pellet was washed with aqueous ethanol (50%, *v*/*v*), resuspended in 3 mL of Milli-Q water, and freeze-dried. FBZ was then loaded into PEG-MCM NPs, similar to the previously described protocol, to produce PEG-MCM-FBZ.

### 2.6. Cy-5 Grafting on MCM-NH2 and PEG-MCM NPs

Cy-5 was covalently attached to MCM-NH_2_ and PEG-MCM NPs. For this purpose, 30 mg of MCM-NH_2_ and PEG-MCM NPs was suspended separately in 3 mL of DMSO. A Cy-5 solution (3 mg/mL) was prepared using DMSO as a solvent. Then, 1 mL of the Cy-5 solution was mixed with 3 mL of MCM-NH_2_ and PG-MCM NPs suspensions and stirred (500 RPM) at 4 °C under dark conditions. After 24 h, Cy-5-loaded NPs were centrifuged (10,000 RPM, 5 min), washed three times with ethanol and water (3:1), and vacuum-dried for 24 h. The dried NPs (MCM-Cy5 and PEG-MCM-Cy5) were stored at −20 °C in the dark.

### 2.7. Physicochemical Characterizations of the Synthesized Drug Nanoformulations

#### 2.7.1. Dynamic Light Scattering (DLS) Measurements

The mean particle size, polydispersity index (PDI), and zeta potential of MCM, MCM-FBZ, MCM-NH_2_, PEG-MCM, and PEG-MCM-FBZ NPs were measured using the DLS method and Zetasizer instrument (Malvern, UK). Briefly, 100 µg of MCM, MCM-FBZ, MCM-NH_2_, PEG-MCM, and PEG-MCM-FBZ NPs were suspended individually in 1 mL of PBS. The suspensions were then sonicated for 5 min and introduced to the instrument.

#### 2.7.2. Morphology of the Synthesized Nanoformulations

The morphology of the MCM, MCM-FBZ, MCM-NH_2_, PEG-MCM, and PEG-MCM-FBZ NPs was evaluated using a Zeiss Sigma SEM and JEOL JEM-1400 TEM. Briefly, the carbon tapes were placed on aluminum stubs, and empty silicon wafers were placed on the carbon tapes. Next, the samples were mounted on the silicon wafers, coated with gold, and screened by SEM. In addition, the NPs were individually scattered into ethanol using sonication (15 min). A drop of each sample was placed onto a carbon-coated 400-mesh copper grid and allowed to dry. The samples were then imaged at 100 kV using the TEM microscope.

#### 2.7.3. Thermogravimetric Analysis (TGA) and Differential Scanning Calorimetry (DSC) Measurements

The thermal properties and thermal composition of MCM, MCM-FBZ, MCM-NH_2_, PEG-MCM, and PEG-MCM-FBZ NPs were investigated using TGA and DSC analyses. Briefly, 5 mg of each formulation was heated up to 900 °C (10 °C/min) in a TGA alumina crucible using the NETZSCH Simultaneous Thermal Analyzer (STA) 449 F3 Jupiter^®^. The drug loading capacity was then measured by comparing the weight loss, where the changes in the weight loss of drug-loaded NPs due to increasing the temperature were recorded, and the final weight was subtracted from the initial weight and considered as the drug loading capacity.

#### 2.7.4. Fourier-Transform Infrared (FTIR) Measurements

FTIR spectra of MCM, MCM-FBZ, MCM-NH_2_, PEG-MCM, and PEG-MCM-FBZ NPs were obtained using a Bruker Alpha-P IR spectrometer (Germany) in the wavenumber range of 400–4000 cm^−1^. For this purpose, the samples were pressed into KBr pellets and then introduced to the FTIR instrument for analysis.

#### 2.7.5. Brunauer–Emmett–Teller (BET) Surface Area Analysis

Nitrogen adsorption–desorption isotherms of MCM and MCM-FBZ NPs were determined using a Micromeritics TriStar™ II 3020 system. The specific surface area was determined by applying the BET method to the isotherm.

### 2.8. Release Study

The drug release behavior of MCM-FBZ and PEG-MCM-FBZ NPs was evaluated at simulated gastric (pH 1.2) and intestinal (pH 6.8) pH levels. Briefly, 4 mg of the NPs (equivalent to 800 µg of FBZ) was individually dispersed in 5 mL of PBS at pH 1.2 and 6.8, respectively, and stirred (200 RPM) at 37 °C. At the time intervals of 0.25, 0.5, 1, 2, 4, 6, 8, 10, and 12 h, 100 µL aliquots of the suspensions were collected and replaced with the equivalent volume of fresh PBS with the same pH. To dissolve the suspended drug, 10 µL of DMSO was added to each collected sample and centrifuged (15,000 RPM, 5 min). The supernatant was then analyzed using a validated RP-HPLC (Agilent HPLC Series 1100) method using Phenomenex Kinetex (C18 100A, 250 mm × 4.60 mm; 5µm), and the amount of FBZ released from MCM and PEG-MCM NPs was measured based on the standard curve of FBZ.

To obtain a calibration curve, 0.5 mg of FBZ was dissolved in 50% (*v/v*) acetonitrile/Milli-Q water, and the mixture was half-diluted six times (seven different concentrations) with Milli-Q water, and centrifuged (15,000 RPM, 5 min). Next, 10 µL of the supernatant was injected into the instrument, and the area under the curve (AUC) was obtained. The calibration curve was then plotted as AUC versus concentration.

### 2.9. Qualitative Cellular Uptake Using Confocal Microscopy

First, 2 × 10^5^ PC-3 cells/well were cultured in RPMI-1640 medium supplemented with 5% (*v/v*) FBS and 1% (*v/v*) penicillin/streptomycin (complete media) in a Cellvis 12-well plate or glass bottom. After 24 h incubation (37 °C, 5% CO_2_), 100 μg/mL of Cy5-MCM and Cy5-PEG-MCM in the complete media were added to the wells and incubated (4 h, 37 °C, 5% CO_2_). All cell experiments were approved by the Research Ethics Committee of the Queensland University of Technology (number 2000000709). The medium was discarded, and the cells were washed three times with chilled PBS (pH 7.4). The cells were fixed using 4% paraformaldehyde in PBS (room temperature, 20 min) and permeabilized using 0.2% triton X-100 in PBS for 30 min. The cells were then incubated with 1% BSA in PBS (1 h, 4 °C) to block nonspecific binding. Subsequently, the cells were stained for filamentous actin using Phalloidin-FITC (40 mM, 20 min) and DAPI (14 mM, 10 min), respectively. In this study, the control was the cells treated only with the medium. After this, PBS was added to prevent dehydration, and the dish was covered with aluminum foil to preserve the stains from the light. Confocal laser scanning microscopy was conducted using a confocal laser scanning microscope (Nikon Air Confocal, Australia) with a 20× objective lens. Excitation of fluorophores was obtained using integrated lasers emitting at 358–461 nm for DAPI and 496–516 nm for Phalloidin-FITC. In addition, Cy-5 signals were detected at 646–670 nm.

### 2.10. Cell Viability

The cytotoxicity effects of MCM-FBZ and PEG-MCM-FBZ NPs were investigated by MTT assay on PC-3 cells, and the results were compared to those of the standard drug. Briefly, PC-3 cells (8 × 10^3^/well) were cultured in the complete media in a Corning^®^ 96-Well TC-Treated Microplate and incubated (37 °C, 5% CO_2_, 24 h). The cells were then treated with FBZ in the standard form, loaded into MCM, and PEG-MCM NPs at the drug concentrations of 6.25, 12.5, 25, 50, 100, 200, and 400 μM. After 48 h incubation (37 °C, 5% CO_2_), the media were removed, and the cells were treated with 100 µL of MTT (0.5 mg/mL PBS, 3 h, 37 °C). To dissolve the formazan crystal, the MTT solution was replaced with 100 µL of DMSO and incubated for 20 min. The absorbance was then read at 570 nm using a microplate scanning spectrophotometer (SPECTROstar Nano), and cell viability was calculated according to the following formula:

$$\%\;\mathrm{Cell}\;\mathrm{viability}=\frac{{\mathrm{Absorbance}}_{\mathrm{sample}}-{\mathrm{Absorbance}}_{\mathrm{background}}}{{\mathrm{Absorbance}}_{\mathrm{negative}\;\mathrm{control}}-{\mathrm{Absorbance}}_{\mathrm{background}}}$$

where the negative control was the treated cells with only the complete media, and the background was only complete media with no cells.

### 2.11. Proliferation Assay

The PC-3 cells (3 × 10^3^ cells/well) were seeded into a 96-well plate. After 24 h, the culture media were replaced with media containing 15.3, 30.6, and 61.2 µM of FBZ standard and loaded into MCM and PEG-MCM NPs. Next, the cell confluency of the treated and untreated cells was determined by the IncuCyte live-cell imaging system (Essen Biosciences, Dandenong South, Vic, Australia) at 10 h intervals for 120 h.

### 2.12. Cell Migration

To evaluate the efficacy of MCM-FBZ and PEG-MCM-FBZ NPs compared to FBZ to inhibit cell invasion, a migration assay was used. Briefly, PC-3 cells were cultured in the complete media in a 6-well plate (CELLSTAR; Greiner Bio-One). When the cells reached 90–95% confluency, scratches were generated on the cell monolayer using a 200 µL pipette tip. The cellular debris was gently removed by washing the plate with the complete media, and the scratches were imaged at 0 h using an Olympus CKX41SF fluorescence microscope (Olympus, Tokyo, Japan). The complete media were then discarded, and the cells were incubated with the media containing FBZ, MCM-FBZ, and PEG-MCM-FBZ NPs at the drug IC_50_ concentration of 30.6 μM. The cells treated only with the complete media were considered as the negative control. After 24 and 48 h, the created wounds (scratches) were imaged, and the data were analyzed.

### 2.13. Reactive Oxygen Species (ROS) Assay

The intracellular ROS was measured based on the DCFH-DA, as a fluorogenic dye, which measures intracellular ROS activity [23]. For this purpose, PC-3 cells at the density of 8 × 10^3^ cells/well were cultured in a 96-well plate containing the complete media. After 24 h incubation (37 °C, 5% CO_2_) and when the confluency reached 70%, the media were discarded, and the cells were treated with FBZ, MCM-FBZ, and PEG-MCM-FBZ NPs at the drug IC_50_ concentration of 30.6 µM. The cells were then incubated (37 °C, 5% CO_2_) for 6 h; the media were replaced with 100 µL of 20 μM DCFH-DA solution and incubated in the dark for 30 min at room temperature. The cells were then washed three times with PBS, and the fluorescence of the treatment was measured at 485 and 520 nm as excitation and emission wavelengths, respectively.

### 2.14. Statistical Analysis

All statistical analyses were performed using GraphPad Prism software version 8.00 (GRAPH PAD Prism Software Inc., San Diego, CA, USA). Statistical differences were analyzed by one-way analysis of variance (ANOVA), and *p* < 0.05 was considered significant.

## 3. Results and Discussion

### 3.1. DLS, TEM, SEM, PDI, and Zeta Potential Characterizations of the Synthesized NPs

**Figure 1:**
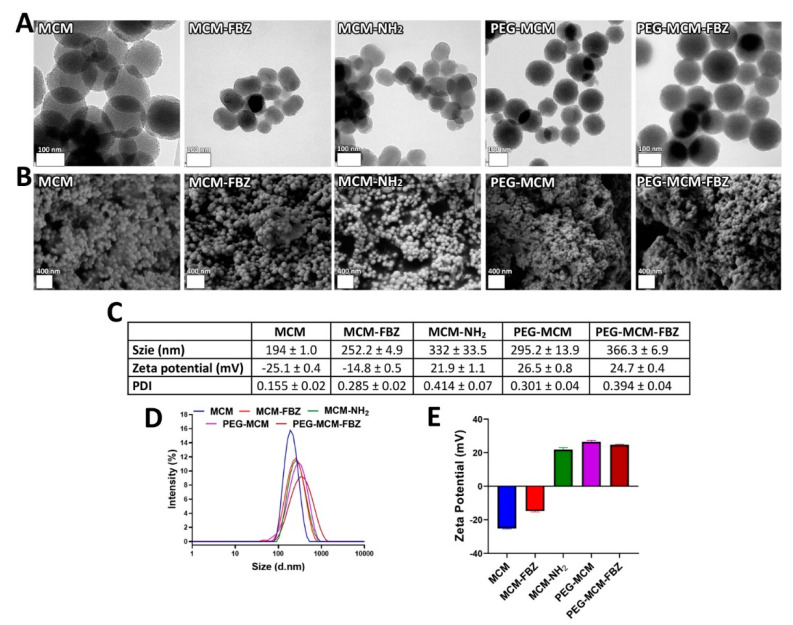
Characterization of MCM, MCM−FBZ, MCM−NH_2_, PEG−MCM, and PEG−MCM−FBZ NPs, in terms of morphology, using (**A**) TEM and (**B**) SEM methods. Results of (**C**,**D**) NP size distribution, (**C**,**E**) zeta potential, and (**C**) polydispersity index (PDI) demonstrate that homogenous and monodisperse NPs were synthesized.

MCM, MCM-FBZ, MCM-NH_2_, PEG-MCM, and PEG-MCM-FBZ NPs were examined by TEM and SEM. As shown in Figure 1A,B, the NPs showed a homogenous and mono-disperse smooth morphology, which is favored over crystalline and irregular particles due to the decreased potential of tissue irritation [24].

The size, PDI, and zeta potential of the new nanoformulations were also measured. The results demonstrated that MCM, MCM-FBZ, MCM-NH_2_, PEG-MCM, and PEG-MCM-FBZ formulations were synthesized in nanoscale dimensions with the size of 194 ± 1.0, 252.2 ± 4.9, 332.0 ± 33.5, 295.2 ± 13.9, and 366.3 ± 6.9 nm, respectively (Figure 1C,D). Moreover, the PDI values of the NPs were found to be in the range of 0.155 to 0.414 (Figure 1C). The particle size and PDI are critical factors to determine the efficacy of NPs as drug carriers [14,25]. NPs with PDI values ranging from 0 to 0.5 are monodisperse and homogenous [26]. Therefore, the DPI values of the synthesized NPs confirmed that they had a homogenous and monodisperse morphology, as indicated by DLS/TEM/SEM measurements. The monodispersity of the drug nanocarriers guarantees their uniform physical, chemical, and biological characteristics for biomedical applications [25]. The zeta potential of the synthesized formulations was measured in the range of −25.1 ± 0.4 to 26.5 ± 0.8 mV (Figure 1C,E). The zeta potential of NPs is critical for determining the formulation stability both in vitro and in vivo. The high zeta potential of NPs (negative or positive) indicates the stability of the drug nanocarriers [27]. In this study, the zeta potential of the synthesized NPs was found to increase from −14.8 ± 0.5 mV (for MCM) to 21.9 ± 1.1 (for MCM-NH_2_), confirming the grafting of the amine groups to the surfaces of MCM-NH_2_ NPs [28]. In addition, the zeta potential increased from 21.9 ± 1.1 mV (for MCM-NH_2_) to 26.5 ± 0.8 mV (for PEG-MCM NPs), indicating the grafting of PEG moieties onto the MCM-NH_2_ NPs. The zeta potential of NPs is a critical factor for determining the formulation stability both in vitro and in vivo, in which, by increasing the zeta potential (negative or positive), the stability of the formulation increases [27]. The high values of zeta potential of the PEG-MCM NPs indicated their good stability as a drug nanocarrier.

### 3.2. TGA and DSC Measurements of the Synthesized NPs

**Figure 2:**
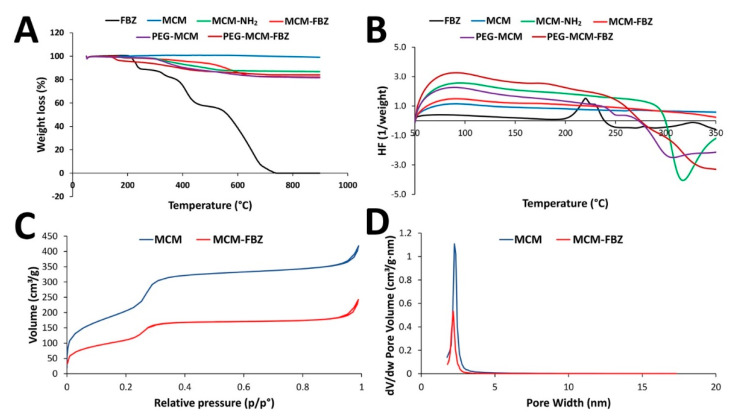
(**A**) TGA thermogram of FBZ, MCM, MCM−FBZ, MCM−NH_2_, PEG−MCM, and PEG−MCM−FBZ NPs; (**B**) DSC thermogram of FBZ, MCM, MCM−FBZ, MCM−NH_2_, PEG−MCM, and PEG−MCM−FBZ NPs; (**C**) N2 adsorption–desorption isotherms, and (**D**) pore size distributions of MCM and MCM−FBZ NPs using the BET method.

FBZ and the synthesized NPs were characterized using TGA analysis (Figure 2A). As indicated by the figure, MCM NPs experienced a weight loss of 1.6 wt.% after heating to 900 °C. This weight loss was attributed to the evaporation of water molecules, indicating the thermal stability and hydrophilic nature of MCM NPs. These properties indicated their suitability for the loading of hydrophobic FBZ drug molecules [29,30]. In addition, the results showed that FBZ started to lose weight at ~212 °C and continued until the drug was completely decomposed at 736 °C. MCM-FBZ NPs demonstrated a weight loss in two steps at 170 and 520 °C, resulting in a weight loss of 18.4%, which was equivalent to the drug loading capacity. The weight loss for MCM-NH_2_ was observed to start at 160 °C and reached a plateau at 550 °C. The total weight loss in these NPs was found to be 13%, corresponding to the presence of the grafted amino group on the NPs’ surfaces. PEG-MCM NPs were found to lose weight between 138 and 900 °C, with a net weight loss of 18%. This weight loss was equivalent to the weight of the amino (13%) and PEG (5%) moieties that were grafted onto the NPs’ surfaces. PEG-MCM-FBZ NPs showed a degradation process at 152 °C, leading to a 17.2% weight loss, which was equal to the drug loading capacity (as explained within the following paragraphs of this article).

DSC analysis (*n* = 3) was performed for the synthesized NPs, where they were heated between 50 and 350 °C at a scan rate of 10 °C/min (Figure 2B). DSC was also used to evaluate the influence of PEGylation on MCM NPs and the drug status inside the NPs. As indicated by the figure, the DSC of FBZ demonstrated a baseline shift at a glass transition temperature (Tg) of 190 °C. Moreover, an exothermic peak was observed at 222 °C, indicating its melting point, which is in agreement with the current literature [31]. However, FBZ did not demonstrate a melting peak in the DSC curves obtained from MCM-FBZ and PEG-MCM-FBZ NPs. The lack of phase transitions, due to FBZ, in the DSC analysis indicated that FBZ is in a non-crystalline state, which, in turn, indicated the competence of MCM NPs to convert the crystalline state of FBZ into amorphous form and, as a result, improved the aqueous solubility and dissolution rate of the drug [14,17]. For MCM, a peak that was observed in the temperature range of 250 to 330 °C indicated an endothermic reaction due to the melting of the NPs. However, there was no T_g_ for MCM NPs in the temperature range of 50–350 °C. PEG-MCM-FBZ NPs demonstrated an endothermic peak at 155 °C, which was attributed to the degradation of PEG moieties (Figure 2B).

### 3.3. BET Surface Area Analysis

N_2_-BET was used to measure the pore size, volume, and surface area of the NPs. Both MCM and MCM-FBZ NPs demonstrated type IV International Union of Pure and Applied Chemistry (IUPAC) isotherms, which are characteristic of MSNPs [32]. Three distinct stages were observed in the MCM isotherm, containing two steps at P/P_0_ = 0.29 and P/P_0_ = 0.34 (Figure 2C). The first stage was attributed to monolayered and multilayered nitrogen adsorption to the mesopore surface of the NPs at relatively low pressures (P/P_0_ < 0.31). The second stage indicated the marked increase in the N_2_ adsorption (0.24 < P/P_0_ < 0.34). Increasing the relative pressure caused an abrupt increase in the amount of N_2_ adsorption due to the capillary condensation within the uniform pores. The third stage in the adsorption isotherm occurred at 0.33 < P/P_0_ < 0.98 owing to multilayer N_2_ adsorption on the external surface of MCM NPs [32]. In addition, the average pore volume of MCM NPs was larger than that of MCM-FBZ NPs (1.10 versus 0.53 cm^3^/g·nm) (Figure 2D). These results confirmed that FBZ was loaded into the MCM NPs.

### 3.4. FTIR

**Figure 3:**
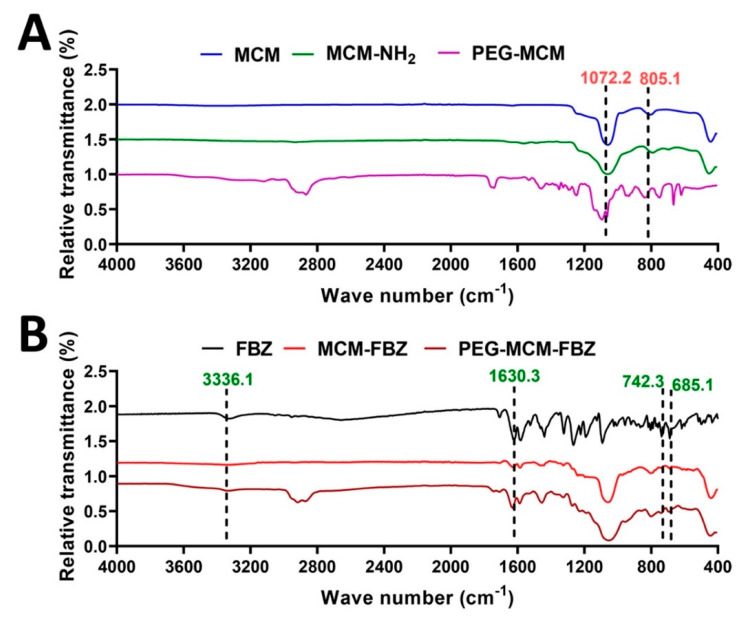
(**A**) FTIR spectrum of MCM, MCM−NH_2_, and PEG−MCM; (**B**) FTIR spectrum of FBZ, MCM−FBZ, and PEG MCM−FBZ NPs.

FTIR spectroscopy was used to determine the chemical structure of FBZ, MCM, MCM-FBZ, MCM-NH_2_, PEG-MCM, and PEG-MCM-FBZ NPs and to confirm the drug loading into the NPs. The bands at the 1072.2 and 805.1 cm^−1^ regions are characteristic of MCM NPs, confirming the synthesis of these NPs [33]. The bands at the 3336.1, 1630.3, 742.3, and 685.1 cm^−1^ regions (Figure 3B) are assigned to FBZ [34]. These bands were also observed in the FTIR of MCM-FBZ and PEG-MCM-FBZ, thus confirming the loading of FBZ into the NPs. The presence of the bands of FBZ in MCM-FBZ and PEG-MCM-FBZ NPs indicated that the drug preserved its chemical structure, and it was loaded into the NPs physically. A minor change in the chemical structure of a drug causes a large change in its physiological activity [35].

### 3.5. Release Study

**Figure 4:**
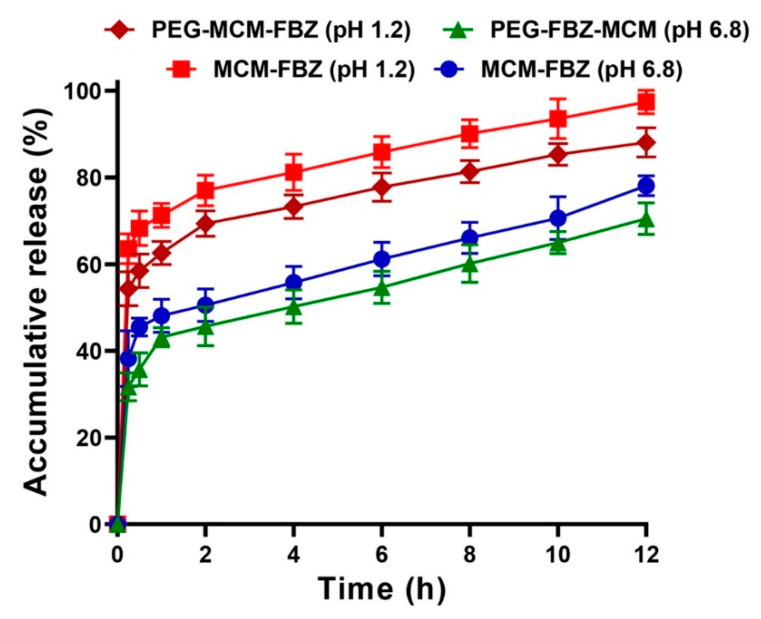
Pattern of FBZ release from MCM-FBZ and PEG-MCM-FBZ NPs, measured at pH 1.2 and 6.8. Statistical analyses were performed using one-way analysis of variance (ANOVA) and F-tests. The data are expressed as mean ± SD (*n* = 3).

FBZ is a poorly water-soluble drug and is administered orally [6,9]. The development of drug delivery systems for the oral administration of drugs is highly recommended due to improving patient compliance and non-invasive administration. In the past few decades, many efforts have been made to develop controlled drug release systems. These systems can regulate drug dissolution from a formulation in a controlled manner [36] and reduce the fluctuations of drug concentrations in the blood. These properties result in a decrease in the drug’s adverse effects [37]. Moreover, increasing the presence time of a short-half-life drug in the plasma using controlled release systems could decrease the administration times [36]. In the present study, the release of FBZ from MCM-FBZ and PEG-MCM-FBZ NPs was measured at pH 1.2 and 6.8 (the pH of the human stomach and intestinal fluids), respectively [38]. As shown in Figure 4, FBZ was released from both NPs in a pH-dependent manner. A burst drug release was observed from both NPs in the first 30 min of the study, in which 65 and 48% of the loaded drug were released from the MCM-FBZ NPs at pH 1.2 and 6.8, respectively. For PEG-MCM-FBZ NPs, 61 and 46% of FBZ were released in the first 30 min at pH 1.2 and 6.8, respectively. This burst drug release from both nanoformulations could be attributed to the release of the absorbed drug onto the NPs’ surfaces [39]. After 30 min, the drug release continued with a mildly increasing trend until 12 h, in which 97 and 78% of the loaded drug were released at pH 1.2 and 6.8, respectively, from MCM NPs. For PEG-MCM-FBZ NPs, 88 and 70% of FBZ were released at the same pH values after 12 h. These results indicated the potency of the NPs to maintain the loaded drug after 12 h incubation at acidic gastric and natural intestinal pH values. The variation in the amount of drug release at pH 1.2 and 6.8 could be due to the effect of pH on the charge of FBZ and MCM NPs as FBZ and MCM NPs could be positively charged at pH 1.2 [40,41].

The identical charge of MCM NPs and FBZ at pH 1.2 caused electrostatic repulsion between the drug molecules and the NPs, resulting in a high rate of drug release [42]. On the other hand, the lower drug release rate from PEG-MCM-FBZ NPs compared to MCM-FBZ NPs could be attributed to the presence of PEG in the structure of the PEGylated NPs. PEG is approved by the Food and Drug Administration (FDA) for human use [43]; it improves the water solubility of the compounds [44,45] and acts as a cover that reduces the drug leakage from the carrier, leading to an increase in the drug stability [45]. PEGylation could also prolong the circulation time of the PEGylated compounds in the body by increasing the hydrophilicity and decreasing the glomerular filtration rate [46]. In addition, the PEGylation of nanomaterials is a promising approach to improve their tumor-targeting efficiency through the enhanced permeation and retention effects [47]. Overall, the results of the drug release study indicated that MCM-FBZ NPs and PEG-MCM-FBZ NPs could release FBZ in a controlled manner, reduce the fluctuations of the plasma FBZ concentrations, and, as a result, improve the FBZ therapeutic effects.

### 3.6. Cell Viability of the Synthesized Nanoformulations

**Figure 5:**
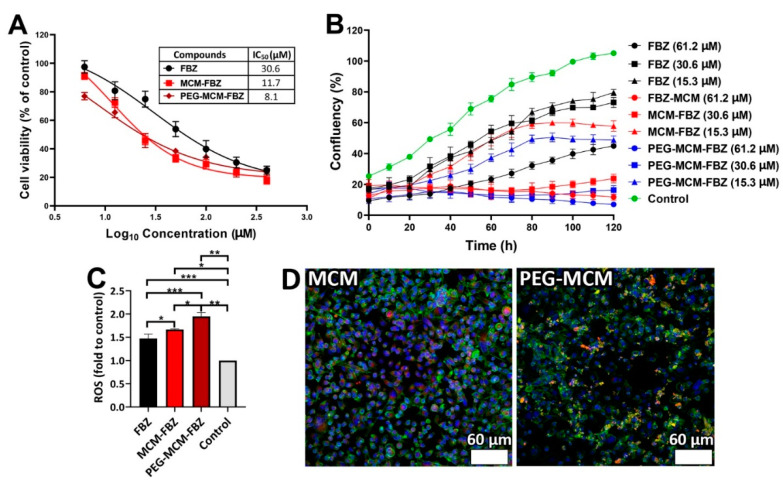
(**A**) Cell viability effects of different concentrations of FBZ, MCM-FBZ, and PEG-MCM-FBZ NPs against prostate cancer PC-3 cells after 48 h incubation. The data are expressed as mean ± SD (*n* = 3). (**B**) ROS generation after PC-3 cells’ incubation with FBZ, MCM-FBZ, and PEG-MCM-FBZ NPs for 6 h. Data are expressed as mean ± SD from three independent experiments and were analyzed using a t-test: * *p* < 0.05; ** *p* < 0.01, *** *p* < 0.001. (**C**) The cell proliferation assay of PC-3 cells in 5 days. (**D**) Fluorescence microscope images of PC-3 cells after incubation with MCM-Cy5 and PEG-MCM-Cy5 NPs for 4 h.

FBZ was loaded into MCM and PEG-MCM NPs to improve the cytotoxicity effects of the drug against prostate cancer PC-3 cells. MCM NPs were functionalized with PEG molecules through grafting to TPGS. For this purpose, the cytotoxicity effects of FBZ in the standard and the nanoformulation forms were evaluated (Figure 5A). To determine the safe concentration of MCM and PEG-MCM, the cytotoxicity of MCM and PEG-MCM was assessed at the concentrations of 62.5, 125, 250, 500, and 1000 µg/mL, and the safest concentration was found to be 250 µg/mL (*p* < 0.05). In addition, the results showed that the standard drug and its nanoformulations caused cytotoxicity effects in a dose-dependent manner. MCM-FBZ and PEG-MCM-FBZ were potent to enhance the efficacy of FBZ (Figure 5A); however, the potency of the PEG-MCM-FBZ was more than that of MCM-FBZ NPs (IC_50_ for FBZ, MCM-FBZ NPs, and PEG-MCM-FBZ NPs were 30.6, 11.7, and 8.1 µM, respectively). The higher potency of PEG-MCM-FBZ NPs could be attributed to the release profile of this formulation, as it could preserve a higher amount of FBZ for an extended period of time [46]. Moreover, covering the carrier by PEG molecules causes a reduction in the drug leakage from the carrier and, as a result, an increase in the drug stability [45]. This, in turn, increases the presence time of the drug in the environment, resulting in an increase in the drug efficacy. Moreover, PEG-MCM-FBZ NPs could produce a high amount of ROS, which can result in higher cytotoxicity [48].

The cell viability effects of different concentrations of FBZ, MCM-FBZ, and PEG-MCM-FBZ NPs were also measured after the incubation of the nanoformulations with prostate cancer PC-3 cells for 48 h. As shown in Figure 5A, all the formulations induced cell cytotoxicity in a dose-dependent manner. However, the cytotoxicity effects of FBZ increased by loading the drug into the NPs. This increase was more prominent in the case of PEG-MCM-FBZ NPs compared to MCM-FBZ NPs.

### 3.7. Proliferation Assay

To determine the effects of FBZ, in its standard and nanoformulation forms, on the proliferation and survival of PC-3 cells, the cell proliferation assay was performed. As shown in Figure 5B, FBZ, MCM-FBZ, and PEG-MCM-FBZ NPs caused the inhibition of cell proliferation in a concentration-dependent manner. In addition, it was found that the potency of FBZ to inhibit the proliferation of PC-3 cells increased when loading into MCM NPs. However, the highest inhibitory effects were observed when the cells were treated with the PEG-MCM-FBZ NPs. These results were in agreement with the results of cell viability.

### 3.8. ROS Assay

Increasing the intracellular production of ROS is a critical factor to suppress the proliferation and induce the apoptosis of cancer cells [48]. Most chemotherapeutics can cause an increase in the intracellular concentrations of ROS [49]. The overproduction of ROS blocks efflux pumps in multidrug-resistant (MDR) cancer cells and sensitizes the cells to chemotherapeutics, leading to more MDR cells’ death [50,51]. In the present study, the potency of FBZ, MCM-FBZ, and PEG-MCM-FBZ NPs to produce ROS in PC-3 cells was evaluated after treating the cells with the NPs for 6 h. As indicated by Figure 5C, all the NPs caused an increase in ROS levels as compared to the control cells by 1.5-, 1.7-, and 1.9-fold for FBZ, MCM-FBZ, and PEG-MCM-FBZ NPs, respectively. The treatment of the cells by PEG-MCM-FBZ NPs produced more ROS than MCM-FBZ NPs by 1.2-fold, indicating the higher potency of this formulation. This increase could be due to the higher potency of PEG-MCM-FBZ NPs to preserve the drug and release it for an extended time as compared to the MCM-FBZ NPs.

### 3.9. Cellular Uptake of FBZ Nanoformulations

The efficiency of cellular uptake of MCM-FBZ and PEG-MCM-FBZ NPs was investigated qualitatively using prostate cancer PC-3 cells and confocal microscopy. Figure 5D depicts the fluorescence microscope images of the PC-3 cells after incubation with MCM-Cy5 and PEG-MCM-Cy5 NPs for 4 h. As indicated by the figure, higher fluorescence intensity was observed from the PC-3 cells after their treatment with MCM-Cy5 NPs as compared to that received from PC-3 cells treated with PEG-MCM-Cy5 NPs. These results indicated that MCM NPs were more internalized into cells when compared to the PEG-MCM NPs. The low cellular uptake of the PEG-MCM NPs could be due to the inhibition of their uptake into the cells by the PEG moieties. PEGylation of NPs can reduce their nonspecific interaction with the blood serum proteins in vitro and, as a result, cause a decrease in the cellular uptake of the NPs. Decreasing the cellular uptake of PEGylated NPs causes an increase in their retention times within the blood circulation [52].

### 3.10. Cell Migration

**Figure 6:**
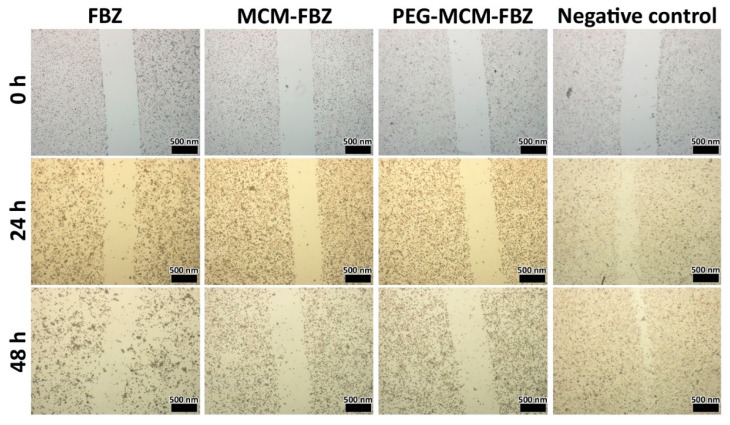
Effects of FBZ, MCM-FBZ NPs, and PEG-MCM-FBZ NPs on prostate cancer PC-3 cells’ migration and invasion.

Abnormal cell migration is an essential component of cancer cell metastasis [53]. Metastasis, in turn, causes more than 90% of cancer-related deaths [54]. For this reason, the efficacy of MCM and PEG-MCM NPs to improve the inhibitory effects of FBZ against the migration of PC-3 cells was evaluated. PC-3 cells were cultured, scratched, and incubated with the IC_50_ concentration of FBZ in the standard form and loaded into MCM and PEG-MCM NPs. The images in Figure 6 show that all formulations inhibited cell migration in a time-dependent manner. However, PEG-MCM-FBZ NPs were more potent to inhibit cell migration when compared to other formulations, indicating the higher efficacy of this formulation over other FBZ formulations, meaning that PEG-MCM-FBZ NPs were more potent compared to other formulations in inhibiting the PC-3 cells’ metastasis. These results were in agreement with the results of the cell viability tests, where PEG-MCM-FBZ NPs were more potent to decrease the prostate cancer cell viability, when compared to FBZ and MCM-FBZ NPs.

## 4. Conclusions

Owing to their promising properties, such as modifiable pore size and volume and easy surface modification, MSNPs have been broadly utilized for the controlled delivery of drugs. In this study, amine-functionalized MCM NPs were synthesized and PEGylated by grafting to TPGS. In addition, PEG-MCM-FBZ NPs with high drug loading capacity (17.2%) were developed. This formulation, compared to MCM-FBZ NPs, demonstrated higher potency to preserve the drug at both gastric and intestinal pH values (65 and 48% from non-PEGylated and 61 and 46% from PEGylated NPs in the first 30 min). In addition, the cell viability and cell migration measurements showed that PEG-MCM-FBZ NPs were more potent to inhibit cancer cell viability when compared to FBZ and MCM-FBZ NPs (IC_50_ was 8.1, 11.7, and 30.6 µM for PEG-MCM-FBZ NPs, MCM-FBZ NPs, and FBZ, respectively). Moreover, PEG-MCM-FBZ NPs caused higher ROS production when compared to FBZ and MCM-FBZ NPs, (1.3- and 1.2-fold, respectively). Accordingly, MCM-41 NPs were found as a promising carrier for improving the anticancer properties of FBZ. Finally, the results pave the way for an in vivo study to confirm the potency of PEG-MCM-FBZ NPs for the treatment of prostate cancer.

## Data Availability

Not applicable.
